# The Tooth Brush; Does It Help or Hinder Oral Cleanliness?

**Published:** 1915-12

**Authors:** 


### THE TOOTH BRUSH; DOES IT HELP OR HINDER ORAL CLEANLINESS?

---

Much discussion has resulted from an article by Dr. B. Feldman, which appeared in *Oral Hygiene,* upon the subject of the tooth brush. The *Literary Digest* asks, under the heading, "The Tooth Brush Indicted":

"Must we unlearn all that our diligent parents and teachers once did their best to instil? If there ever was an implement generally acknowledged as indispensable to cilivization, it was surely the tooth brush. It has been to most of us almost a religious symbol of that personal cleanliness which the old Law places next to Godliness. And now, forsooth, we are told that it is not conformable to modern hygiene and sanitation! Its sins are both of omission and commission. It fails to remove impurities, and it serves as an efficient instrument to inoculate the teeth and gums with disease. Thus it has served as an instrument, not of personal hygiene, but of infection."

The following extract from the article in question will serve to indicate the position taken by Dr. Feldman:

"Not only has the public become accustomed to look upon the brush as necessary, but our teachers and the great army of dentists are recommending its diligent use. This teaching of school children and of adults how to use the brush properly constitutes what I consider 'the menace of the tooth brush'; because it has been proved to me that the brush is defeating the very purpose of our oral hygiene movement and that we are actually infecting the mouth instead of cleaning it by the use of the filthy, germ-ridden thing. Dr. Head called the attention of the profession to the dirty condition of the brush as it is used by the general public. Professor Miller proved that the brushing action of the bristles upon the surfaces of the teeth had a very injurious mechanical wasting effect near the necks of the teeth. Professor Hutchinson reported the conclusions which were reached in this matter by research workers, and his remarks are so emphatic that the matter can not be well ignored. The plain truth is that the brush is a dangerous instrument which is practically impossible to sterilize. It can not be boiled with impunity, and practically all agents, such as tricresol or formalin, render the bristles of the brush or the handle unfit for further use. To quote Professor Hutchinson: 'Not only the public, but the dentists themselves, have little conception of the filthy state of the comparatively clean tooth brush as used in every-day life.'

"But granting the impossible—*i. e.*, that the brush with its bristles covered with a thin ribbon of tooth paste or powder is sterile—why should we use it when it does not reach the interproximal spaces where it is most important that the bristles should reach? Tooth decay starts in these spaces in the majority of cases. An efficient cleaning is probably never obtained by the brush. What is more probable is that many of the germs that are present on the bristles are deposited in these spaces. The silk floss does reach between the teeth and does clean out the food debris. It seems self-evident that the brush fails to do what it is supposed to do, so why use it when it does not do any good?

"To cite an example which was given to me by a friend a few minutes before I gave an oral hygiene talk to school children: the big brush that is used by the street cleaners will clean the surfaces of the cobblestones in the gutter, but will glide over the cracks where most of the dirt is settled. This seems to me to be a splendid word-picture; and its worthy object was to illustrate how and why to use the tooth brush to dislodge the food debris 'between the cracks.'

"But why should we follow the example or pattern the cleaning of teeth after the crude method of the cleaning of gutters having cobblestones? To cite other examples: stiff brush with a liberal amount of soap and water, vigorously applied, will clean the smooth surfaces of floors; the cloth of a person's suit can be cleaned by the clothes brush and one's shoes can be polished by a shoe brush. Inert substances can not cry out that this rubbing *hurts*. Dr. W. D. Miller proved that the same kind of agent, a brush of smaller size but exactly the same in principle, *does hurt* the soft tissues of the oral cavity. This tearing and rubbing on the gums of the teeth is done by a brush which is filthy with those very germs that we are so very anxious to rid the mouth of. Would the surgeon sanction the cleansing of an open wound with an infected brush which was covered with an antiseptic tooth paste or powder? Are we oral surgeons, therefore, justified in teaching children and adults to use such an instrument on soft gums and teeth? Experiments were made which proved that the brush contains a quantity of germs comparable with the number of germs found in sewage. Twelve sterile brushes were used in these experiments, applied once on the teeth, rinsed ten times in a tumbler of water, were left to stand for twelve hours, when all the bristles were removed with sterile forceps and the organisms counted in the usual way. In eight cases out of twelve the results were as quoted. One hates to think how filthy the brushes are that are used daily, especially by those people in whose mouths septic processes are taking place. No one that can look squarely at facts and that has the courage to stand by a proved principle can continue to use the brush nor advise its use for his clientele. . . .

"Our research workers, of which we have far too few, have proved conclusively, to me, at least, that the tooth brush is undesirable and inefficient. It has been shown that pastes and powders and lotions are beneficial, whenever they do not discolor the teeth. Of what good is research work if the rank and file do not benefit by the findings? The conclusion which I have reached is that an able and unbiased board or commission of dentists should solve this problem for the dental profession, and give us a technique for cleaning the oral cavity that is *real oral hygiene*. This could then be taken up by the rank and file, and the doctrine spread broadcast. Until such method is adopted, may I suggest that we go back to the old Japanese method of using the clean forefinger to massage and clean the gums and outer surfaces of the teeth? It seems to be Nature's own instrument that 'just fits the bill.' Instead of using salt and water as did the Japanese, we can use our modern lotions, to be followed by the recognized efficient silk floss or strips. Mind you, this is my own idea; but I cite it only to create a discussion among dentists to obtain *real oral prophylaxis*. But let us start right by abandoning the filthy tooth brush once for all."

*The Washington Times* refers to Dr. Feldman's article as "The Tooth Brush's Peril," and comments in a recent issue as follows:

"These scientific gentlemen stop at nothing. They do not hesitate to take sure aim at the things we take most for granted. Long ago we grew used to their attacks upon religion, homes, and other recognized institutions, but even at that it is something of a shock to have one of them take a fling at the very bulwark of our civilization—the tooth brush.

"Even the small boy has regarded the implement as a sad but necessary evil. Yesterday found thousands of children marching to New York's public schools with brushes neatly wrapped in sterilized paper, much as the child of former years went to Sunday School displaying a Bible earned by a year's unremitting attendance. Irksome as the tooth brush is no one has had the temerity to attack it, hitherto, even when grown up. Many adults remembered other indignities of early days, and there are some who aver that spanking should be abolished, and many mourn because that reform was introduced too late to help much.

"This scientist's knowledge of human nature is faulty. What if the crevices of the molars are not reached by the bristles, which he pictures teeming with living and pernicious microbes? Would he rob the great majority of persons of the mental satisfaction that comes from the regular use of the prophylactic implement? Would he deprive them of that glow of satisfaction, that feeling of innate superiority, that subtle class distinction that arises from the assiduous practice of this simple ceremony?

"The tooth brush will die hard. After carrying in into heathen lands like a veritable mace of progress, after waving it at the submerged tenth as the mystic sign of uplift, after flaunting it before school children as the magic key to sanitation and hygiene, it will not so easily be lost on the say-so of any literal, plodding scientist."

An Alabama daily states that it is not impressed with

Dr. Feldman's "denunciations of the tooth brush," and proceeds:

"Not only the dictates of common sense, but the views of the best known dental surgeons in the country stand in opposition to the 'findings' of Dr. Feldman.

"If the street sweeper's brush will glide over the cracks in the cobblestones, cleaning only the surface, why go to the great pains and expense incident upon sweeping the streets of our cities? Why not abandon this plan of cleaning and use wet blankets to mop up the dirt and filth?

"Dentists admit that a perfect apparatus for cleaning the teeth has not yet been devised; the ideal tooth brush is yet to be found; but any tooth brush is better than none, and most tooth brushes get excellent results when used systematically and intelligently."

The *Cincinnati Post* treats the matter editorially, in a humorous vein, under the caption, "The Lair of the Germ:"

"Ah! what's the use of combing your hair, swatting the fly, sleeping on a roof, lambasting the baby for eating mud pies? These germologists never agree on anything for five days, anyhow!

"The tooth brush, says Dr. Feldman, is full of germs and cannot be cleared of germs by any ordinary process. On every bristle of the tooth brush there are 74,198 germs, which, in two weeks or thereabouts, will produce, by geometrical progression, 798,000,000,084 more germs. These latter, in turn, as the tooth brush ages, will produce progeny to the number of—well, we just haven't type enough to show you that number. Call it plenty enough to scare the daylights out of you.

"Moreover, according to Dr. Feldman, the tooth brush wears away your teeth and hurts your gums, and folks should go back to the old Japanese method of cleaning their teeth with the forefinger, rather than risk the common tooth brush.

"However, cleaning teeth with a forefinger, like a Jap, or with a big toe, like a hen, won't appeal to many. We'll stick to the tooth brush until the germ sharps get up some sort of a buzzer that we can take to the washstand and buzz the germs galley west with it."

The serious side of the question from the standpoint of the advancement of the oral hygiene movement is illustrated by the comment of the *Denver News.* After referring to the recent "Tooth Brush Week," when the children of the New York public schools received instruction in the proper care of the teeth and were given practical demonstrations in the use of the tooth brush, this paper says:

"A more serious question is raised by Dr. Bernard Feldman. The doctor indicts the tooth brush as a peril rather than as a means of conserving the teeth. There are three counts in his indictment. First, it fails to reach the crevices of the teeth where organic matter lodges; second, it injures the surface of the teeth by too harsh a friction, and, third, it deposits more germs of disease than it removes. Dr. Feldman advises the use of silk floss.

"If the tooth brush is as dangerous as he says, we ought to know it before the movement for tooth brush drills spreads through the schools of the country. We confess to a sense of shock at his charges. It is like hearing an old and trusted friend accused of treachery."

A New York paper refers to this "condemnation of the tooth brush," and assuming it to be merited, proceeds, on its own account, to give some advice to the public how to clean the teeth without the aid of a brush. To quote: "It is wise to *dip the cloth which acts as a substitute for the brush* in some powder sufficiently coarse *to produce some grinding effect* . . . Dental floss is a splendid material for substituting tooth brushes."

These comments are published by *Oral Health* to show the widespread public interest taken in the problem of individual mouth cleanliness and the public oral hygiene campaign. This movement has assumed nation-wide proportions and has obtained the recognition and support of health boards, school authorities and public bodies everywhere.

It is no doubt true, as Dr. Feldman suggests, that the dental profession is somewhat at sea in discussing preventive measures. However, one cannot but doubt the wisdom of condemning and recalling the modern tooth brush until there is some degree of unanimity in the profession itself regarding some better article or method for accomplishing the desired result. However, the article will accomplish much in disturbing the quiet slumbers of many dentists who are satisfied to let "the other fellow" do their thinking, and will certainly be a stimulus to the further study of modern methods of oral hygiene and prophylaxis.—*Oral Health.*

